# Enhanced giant magnetoimpedance in heterogeneous nanobrush

**DOI:** 10.1186/1556-276X-7-506

**Published:** 2012-09-10

**Authors:** Yi Zhang, Congpu Mu, Caiqin Luo, Juan Dong, Qingfang Liu, Jianbo Wang

**Affiliations:** 1 Institute of Applied Magnetics, Key Laboratory for Magnetism and Magnetic Materials of the Ministry of Education, Lanzhou University, Lanzhou 730000, People’s Republic of China

**Keywords:** nanobrush, giant magnetoimpedance, micromagnetic simulation, FMR

## Abstract

A highly sensitive and large working range giant magnetoimpedance (GMI) effect is found in the novel nanostructure: nanobrush. The nanostructure is composed of a soft magnetic nanofilm and a nanowire array, respectively fabricated by RF magnetron sputtering and electrochemical deposition. The optimal GMI ratio of nanobrush is promoted to more than 250%, higher than the pure FeNi film and some sandwich structures at low frequency. The design of this structure is based on the vortex distribution of magnetic moments in thin film, and it can be induced by the exchange coupling effect between the interfaces of nanobrush.

## Background

Giant magnetoimpedance (GMI) effect has been considered as a potential physical effect that may take the place of giant magnetoresistance (GMR) because of its higher field sensitivity and better signal intensity [1]. Since GMI changes as the function of external dc (direct current) magnetic field or applied dc/ac (alternating current) currents, it is possible to design GMI-based sensors that can measure either magnetic fields or dc/ac currents [2,3]. Based on the applied stress dependence of the GMI effect, new kinds of stress sensors have been proposed [4-6]. Many industrial and engineering applications of GMI sensors have been proposed and realized to date, including traffic controls, automobile uses, biomedical sensors, and so forth [7-9]. Typical materials which may give rise to GMI effect are amorphous wires, amorphous ribbon, multilayer films, and other soft magnetic materials [10-15]. Normally, the diameter of amorphous wires and the thickness of ribbons are up to micrometer scale, and the multilayer films with a high-GMI ratio work in gigahertz. In recent years, with the rapid development of nanomaterials, the size of magnetic sensors is projected to reach nanoscale. However, traditional GMI materials do not satisfy the desired size, and it is a challenge to find new kinds of nanomaterials, which can have both an obvious GMI effect and a rapid magnetic response at a low frequency.

GMI effect is normally attributed to a combination of skin effect and high sensitivity of transverse permeability to the external dc field [16,17]. In a magnetic medium, the skin depth is dependent on the transverse magnetic permeability (*μ*_t_) through $\delta_{m}=c/\sqrt{2\pi f\mu_{t}\sigma }$, where *σ* and *μ*_t_ are the electrical conductivity and the transverse permeability of the ferromagnetic material, respectively. For amorphous ribbons and wires, many approaches have been tried to improve the GMI ratio, such as annealing, ion irradiation, glass coating, and patterning [18-20]. Essentially, all the above approaches to enhance GMI ratio are based on the changes of magnetic domain and induced transverse distribution of magnetic moments [1]. For the films, the sandwich structure is the traditional approach to depress the skin effect and improve the GMI ratio, but the low GMI ratio and high working frequency constitute major negative factors for applications. Obviously, it is urgent to solve the problem of how to induce the transverse moment distribution and enhanced GMI ratio in the nanomaterial.

As a typical nanostructure, nanobrush, has been studied as one of the nanodevices for its special characters [21-23]. However, it is rare as far as the magnetic sensor applications are concerned. In this paper, a kind of magnetic nanobrush, consisted of nanofilm and nanowire array, is prepared. Considering that the origin of GMI is the change of transverse permeability under an external applied field, the design of the structure is based on the different vortex distributions of magnetic moments in thin film, which could be induced by the exchange coupling effect in the interface of heterogeneous nanobrush. To our relief, the magnetic heterogeneous nanobrush shows an excellent GMI ratio and high magnetic response at a low frequency.

## Methods

Self-ordered anodic aluminum oxide (AAO) templates were prepared using the two-step anodization process in an oxalic acid solution (0.3 mol/L). The anodization potential was 40 V (20 V for sulfuric acid, 80 V for phosphoric acid). The process consisted of two distinct steps: in the first one, the high purity (99.9995%) aluminum foil was subjected to the first anodization for 40 min; after the selective removal of the alumina film formed on the surface of the Al anodized foil with an aqueous solution of 1.8% H_2_CrO_4_ and 6% H_3_PO_4_, the Al foil was subjected to the second anodization step for 6 h. Fe_25_Ni_75_ thin films were prepared by RF magnetron sputtering onto the surface of nanowire arrays with common base pressure below 3 × 10^−5^ Pa and processing Ar pressure of 0.4 Pa. The RF power was 140 W, and the time of deposition was 30 min. Moreover, the FeNi film would have to cover the top of AAO template, and the surface of the sample was conductive.

X-ray diffraction confirmed the composition of the nanowires array. Magneto-optic Kerr effect (MOKE) was used to obtain the surface magnetic properties of the composite structure. The surface topography and nanostructure were observed by scanning electron microscope (SEM). Micromagnetic simulations were performed with the three-dimensional object-oriented micromagnetic framework method. The exchange constants of the film and wires were 1.3 × 10^−11^ J/m and 1.75 × 10^−11^ J/m, respectively. The damping parameter *α* was 0.5, the mesh size was 5 × 5 × 5 nm^3^, and the saturation magnetization of permalloy film and Fe nanowires were 8.6 × 10^5^ and 1.71 × 10^5^ A/m, respectively. The ferromagnetic resonance was performed using an X-band spectrometer (JES-FA300; *f* = 9 GHz; JEOL Ltd., Tokyo, Japan). Before magnetoimpedance (MI) measurement, the samples were tailored into small pieces with length of 20 mm and width of 3 mm. An impedance analyzer (Agilent 4294A; Agilent Technologies Inc., CA, USA) was used in the four-terminal contact mode to measure the impedance (*Z*). All the electronic instruments were controlled using LabVIEW.

## Results and discussion

**Figure 1:**
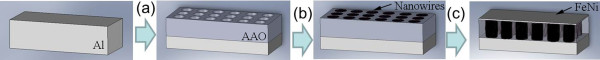
**Preparation of the heterogeneous nanobrush based on the AAO templates and magnetron sputtering. **(**a**) A regular AAO template was achieved by two-step oxidation; (**b**) Fe, Co, or FeCo alloy nanowires were deposited by alternating current; (**c**) a nanoscale film covered the surface by magnetron sputtering (the AAO template was hidden in this sketch).

It is well known that the AAO templates are widely used for the preparation of nanowires [24,25]. Figure 1 shows the preparation of the heterogeneous nanobrush based on the AAO templates and magnetron sputtering. To prepare the nanobrush, high purity aluminum was annealed to remove the internal stress and then polished via electrochemical means. As shown in Figure 1a, various diameters (20, 50, and 100 nm) of the AAO templates were prepared using different electrolytes. Fe, FeCo, and Co nanowires were deposited in these AAO templates by ac electrode position at room temperature, as shown schematically in Figure 1b. After cleaning, a 100 ± 10-nm thick Fe_25_Ni_75_ film was sputtered on the surface of AAO templates, as shown in Figure 1c.

**Figure 2:**
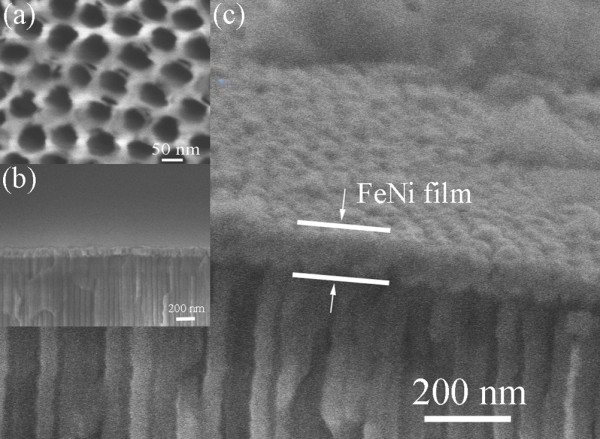
**Surface topography of AAO templates and the cross section of the nanobrush. **(**a**) AAO templates with diameters of 50 nm; (**b**) and (**c**) are the profile of the nanobrush with 20 and 50 nm nanowire array.

The AAO templates are used to fabricate the nanobrush, and its cross profile was revealed from the microscopic investigations. SEM image of the self-ordered AAO templates is taken in top view (Figure 2(a)). The uniform SEM contrast observed from the side (Figure 2(b) and (c)) proves the homogeneous Co deposition inside the nanowires of the whole AAO templates and along their whole length.

**Figure 3:**
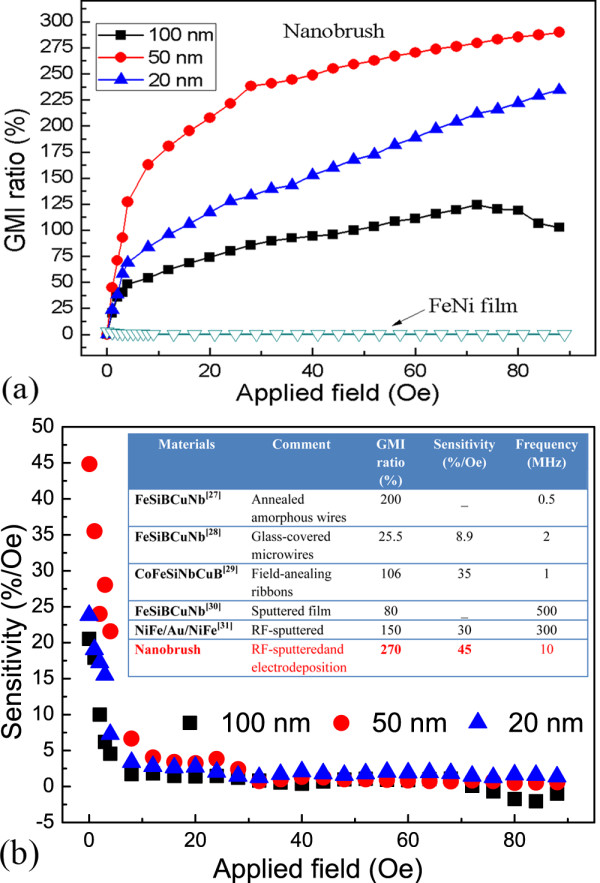
**GMI ratio and magnetic response of the nanobrush. **(**a**) GMI ratio of the nanobrush combined with Co nanowires with different diameters at a frequency of 10 MHz; (**b**) magnetic response of the nanobrush with different diameters of nanowires.

Figure 3 shows the field dependence of GMI ratio of the nanobrush combining FeNi film and Co nanowires with different diameters at the frequency of 10 MHz. As the external applied field increases, the GMI ratio also increases, and an obvious change of GMI ratio takes place at the small field. The diameter of 50 nm shows the best GMI ratio, which is more than 270% under 80 Oe field. The GMI ratios of the nanobrush with 100 and 20 nm nanowires are 120% and 90%, respectively. A huge enhancement of the GMI ratio can be observed from FeNi film to nanobrush. The GMI ratio with applied magnetic field can be expressed as Δ*Z* / *Z* = *Z*(*H*_ex_) − *Z*(*H*_0_)] / (*H*_0_)*100%, where *Z*(*H*_ex_) and *Z*(*H*_0_), respectively, represent the impedance in a magnetic field *H* and without field. The GMI curves agree well with a typical characteristic, which can be explained by the magnetization rotation model [26]. The transverse magnetic permeability related to the GMI ratio increases with increasing applied field, and it reaches a maximum value when the applied field is equal to the anisotropy field of the film. Thereafter, the permeability decreases with field, and the GMI ratio gradually drops. However, it should be noted that the field dependence of the GMI ratio differs a little from the situation of the normal film. Traditionally, as a kind of soft magnetic material, FeNi film could be saturated with a field under 100 Oe, and the permeability will decrease. But for the film in nanobrush, saturation field will reach up to more than 400 Oe; the permeability will not drop dramatically when the field is 400 Oe. So, the GMI ratio could not be decreased until the field is up to the saturation field. Besides, due to the exchange coupling effect introduced by the porosity of magnetic nanowire array, heterogeneous nanobrush shows excellent GMI ratio. The coupling effect of FeNi film and Co nanowires leads the magnetic moments to transverse direction in an applied field. The diameter of nanowires plays an important role in this phenomenon. Fifty nanometers is slightly less than the critical size of single domain of nanowires, so the sample shows the best GMI ratio. For the nanobrush with small diameters, it has small contact area between the nanowire array and FeNi film. On the contrary, when the diameter is large enough, multidomain structure of nanowires might reduce the exchange coupling effect and prevent the transverse permeability.

It should be emphasized that not only the GMI ratio but also the magnetic response is important for high-performance sensor application. Figure 3b shows the magnetic response to the different compositions of nanowires. The sensitivity (*S*) of the GMI was defined as follows: *S* (%/Oe) = (ΔZ / Z) / ΔH, where ΔH is the change of the magnetic field. At very small external applied field, the field sensitivities of GMI effect of the nanobrush are 25%/Oe, 20%/Oe, 45%/Oe. Afterwards, it begins to decrease and display a value which is approximately equal to zero. The inset table shows the GMI ratios and sensitivities of several typical materials. The optimal structure and size is the FeNi film with 50-nm Co nanowire arrays which show a higher GMI ratio and more sensitive response than these amorphous wires, ribbon, and NiFe/Au/NiFe sandwich structure [27-31].

**Figure 4:**
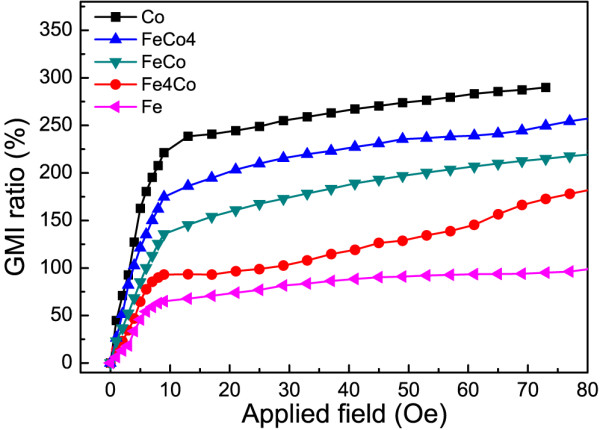
GMI ratio of nanobrush using nanowire arrays with different compositions at a frequency of 10 MHz.

The exchange coupling can also be influenced by the composition of nanowires. Figure 4 shows the magnetic field dependence of the GMI ratio of nanobrush fabricated by nanowires with different Fe and Co composition. The Fe/Co ratio in sample name is the ion concentration ratio in electrolyte. It has been measured at the frequency of 10 MHz. The diameters of all nanowires are 50 nm. The GMI ratios of nanobrush are raised up to more than 200%, which will continue to enlarge if the applied field increases. The GMI ratios are improved by increasing Co content, leading to a stronger coupling effect.

**Figure 5:**
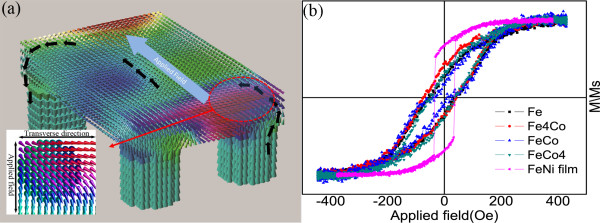
**Micromagnetic simulation of the top surface magnetic properties of the nanobrush. **(**a**) The distribution of static magnetic moment for nanobrush by micromagnetic simulation; (**b**) the top surface magnetic properties of nanobrush measured by NanoMoke.

Some models could qualitatively interpret basic GMI features over abroad frequency range, and they were just fit for cylindrical and planar magnetic conductors [26,32,33]. For the nanobrush structure, it is hard to calculate the impedance and transverse permeabilities by solving simultaneously the reduced Maxwell equation and Landau-Lifshitz equation for the motion of the magnetization vector. Here, in order to directly observe the transverse moment distribution of film caused by exchange coupling effect, micromagnetic simulation is used to show the distribution of magnetic moments of nanobrush (Figure 5a). The nanobrush combined with permalloy film and cubic Fe nanowires is used in simulation. The thickness of the permalloy film and the diameter of Fe nanowires are 50 nm. An external field applied in the plane of the film is 100 Oe. The direction of magnetic moment is denoted by the arrows, and the colors of the arrows show the distribution of moments with different components of transverse moments. As we know, the magnetic moment of the film lies in the plane for its shape anisotropy, and the magnetic moments of nanowires are along the long axis of wire for the same reason. Strong exchange coupling effect happened on the interface of nano film and nanowire array, leading to a vortex distribution of magnetic moment. Thus, the GMI effect will be enhanced due to the transverse component magnetic moments. The inset shows the top view of the moment distribution of the cross section of the film and nanowires. Due to exchange coupling effect, lots of moments turn to be perpendicular to the applied field. This phenomenon can be proved by the magnetic hysteresis loop of top surface of nanobrush. As shown in Figure 5b, the soft magnetic FeNi film is easy to be magnetized to saturation at a field of 100 Oe. However, when there are nanowire arrays combined to form into a nanobrush, the saturation field turns from about 100 to 400 Oe, and the coercivity of the nanobrush’s surface FeNi layer is larger than pure FeNi film.

**Figure 6:**
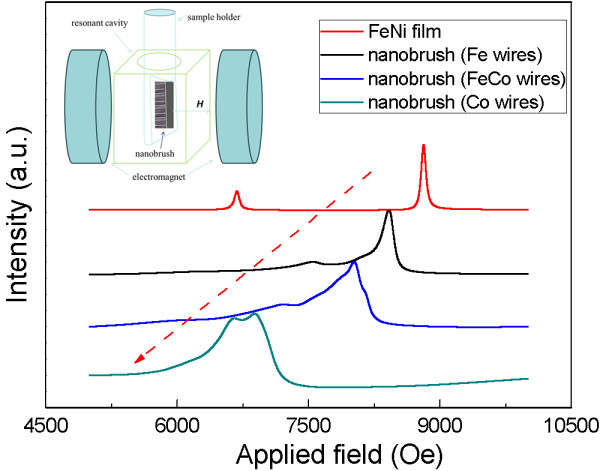
FMR spectra of nanobrush when the applied field was perpendicular to the top surface of the nanobrush.

Ferromagnetic resonance (FMR) has proven to be one of the ideal techniques to study magnetic anisotropies and dynamics. This is because the resonance field and linewidth values are very sensitive to properties such as surface and magnetic interactions between different elements at the interface. As mentioned above, the transverse distribution of magnetic moments can be induced by exchange coupling effect, which can also be proved by the FMR of the top surface of the nanobrush [34,35]. In order to reflect the difference of exchange coupling effect, Figure 6 shows the FMR at *f* = 9 GHz as a function of the field applied out of the plane of FeNi film and films composited with nanowire arrays (shown in the inset of Figure 6). The resonance field of single FeNi film is lower than 9,000 Oe [36], and the FMR peaks of the nanobrushes drift to low field with the increase of Co. Because the Co nanowire array has the strongest exchange coupling effect between the interface, the effect tends to lead the moments to be perpendicular to the film and decreases the resonance field. So, the peak turns left and moves to about 7,000 Oe. For the samples with different composition, the broadening linewidth of FMR peaks is caused by different exchange coupling effects between the interfaces. The exchange coupling effect may increase the transverse permeability, which corresponds well to the results of GMI effect in Figure 4.

## Conclusions

A new kind of exchange coupling effect is used to enhance the transverse moment distribution. A surprising GMI ratio and magnetic respond of heterogeneous nanobrush are found in the experiment. The diameter of the nanowires has obvious influence on the GMI characters of the nanobrush. The sample combined with 50 nm Co nanowires and Fe_25_Ni_75_ film shows the best GMI ratio and sensitivity, which could reach 270%/Oe and 45%/Oe, respectively. The phenomenon can be explained by the obvious distribution of transverse magnetic moments, which is induced by the exchange coupling effect between the interface of nanowires and film, and the exchange coupling effect can be regulated by the content of Co element of nanowire arrays. Micromagnetic stimulation shows the distribution of magnetic moments when the nanowire array act on the soft magnetic film. Out-of-plane FMR result confirms the different coupling effects of the different nanobrushes, which corresponds well with the GMI results of the nanobrushes.

## Competing interests

The authors declare that they have no competing interests.

## Authors’ contributions

YZ, JD, and CL did the study of the optimum conditions of the nanobrush in giant magnetoimpedance effect. YZ wrote the main part of the manuscript. CM established the theoretical formalism and participated in the micromagnetic simulation. QL and JW supervised the whole study. All authors discussed the results and implications, and commented on the manuscript at all stages. All authors read and approved the final manuscript.

## Authors’ information

JW and QL are professors of the Institute of Applied Magnetics, Key Laboratory for Magnetism and Magnetic Materials of the Ministry of Education, Lanzhou University. YZ and CM are Ph.D. students.
